# Curve analysis of tissue oxygen desaturation after a venous occlusion test does not identify the central venous hemoglobin oxygen saturation

**DOI:** 10.1186/cc10864

**Published:** 2012-03-20

**Authors:** G Friedman, C Alan, A Meregalli, A Lima, J Bakker

**Affiliations:** 1 UFRGS, Porto Alegre, Brazil; 2 Complexo Hospitalar Santa Casa, Porto Alegre, Brazil; 3 University Medical Center Erasmus, Rotterdam, the Netherlands

## Introduction

We aim to compare the time for the equivalence of tissue oxygen saturation (StO_2_) with central venous hemoglobin oxygen saturation (ScvO_2_) measured at depths of 15 and 25 mm.

## Methods

Twenty-one critically ill patients were included. The ScvO_2 _was measured by blood gas analysis. Thenar StO_2 _was continuously monitored (Model 650 InSpectra Tissue Spectrometer; Hutchinson Technology Inc., MN, USA) in 15 mm (StO_2- _15) and 25 mm (StO_2- _25) depths. The venous occlusion was performed using an automatic pneumatic device maintaining inflation pressure 10 mmHg above the diastolic pressure. A StO_2 _desaturation curve was plotted to identify the time for equivalence to ScvO_2_.

## Results

Age: 59 ± 17 years, APACHE II score: 21 ± 7, SOFA score: 7 ± 4, ScvO_2_: 75 ± 6%, blood lactate: 1.6 ± 1.2 mmol/l, capillary refill time: 9.1 ± 8.1 seconds, body temperature: 36.7 ± 1.1°C. Measurements were performed for up to three consecutive days (total measurements: 43). In four patients the equivalence was not identified. The curve analysis showed that StO_2 _desaturation time equivalency for ScvO_2 _was greater for StO_2- _15 than for StO_2- _25 (88 ± 54 seconds vs. 79 ± 56 seconds, *P *< 0.01). The Pearson correlation index for equivalence times for StO_2- _15 and StO_2- _25 was 0.92 (*P *< 0.001), but Bland-Altman analysis showed a significant difference between the times (mean difference: StO_2__25 - StO_2- _25: -7.9 ± 37.8 seconds). An arbitrary time of 80 seconds identifies the ScvO_2 _in 58% of cases.

## Conclusion

The analysis of the StO_2 _desaturation curve does not adequately identify the hemoglobin central venous oxygen saturation.
